# The changing profile of obstructive sleep apnea: long term trends in characteristics of patients presenting for diagnostic polysomnography

**DOI:** 10.5935/1984-0063.20210005

**Published:** 2022

**Authors:** Ross J. Marriott, Nigel McArdle, Bhajan Singh, Stuart King, Ivan Ling, Kim Ward, Ellie Darcey, Daniela Bond-Smith, Sutapa Mukherjee, Lyle J. Palmer, David Hillman, Gemma Cadby

**Affiliations:** 1 Centre for Genetic Origins of Health and Disease, School of Biomedical Sciences, University of Western Australia.; 2 School of Anatomy, Physiology and Human Biology, University of Western Australia.; 3 West Australian Sleep Disorders Research Institute, Sir Charles Gairdner Hospital.; 4 Adelaide Institute for Sleep Health, Flinders Health and Medical Research Institute, Flinders University, Adelaide, S.A.; 5 Sleep Health Service, Respiratory and Sleep Services, Southern Adelaide Local Health Network, Adelaide, S.A.; 6 School of Public Health, Faculty of Health and Medical Sciences, University of Adelaide, Adelaide, SA, Australia.

**Keywords:** Polysomnography, Obesity, OSA, Sleep Disorders, BMI, Epidemiology

## Abstract

**Introduction:**

We aimed to analyze long-term trends in characteristics of patients undergoing diagnostic polysomnography (PSG) and subsequently diagnosed with obstructive sleep apnea (OSA) to inform delivery of sleep services.

**Material and Methods:**

We studied 24,510 consecutive patients undergoing PSG at a tertiary-care sleep service between 1989 and 2013. OSA was defined by an apnea hypopnea index (AHI)≥ 5 events/hour. Changes to hypopnea definition and flow sensing techniques in 2002 created two distinct AHI scoring periods: American Sleep Disorders Association (ASDA) 1989 - July 2002 and American Academy of Sleep Medicine (Chicago) from August 2002.

**Results:**

Over 23.5 years there was a steady increase in proportion of females (15% to 45%), small increases in average age and BMI, and a small decline in socioeconomic status in the overall group. AHI varied between scoring periods both overall [ASDA 10.8/h (3.2-29.6), Chicago 24.3/h (11.8-48.1)] and in the large subgroup (80.7%) diagnosed with OSA [ASDA 20.7/h (10.6-44.1), Chicago 27.4/h (14.8-51.5)]. OSA diagnosis rates increased in the Chicago period (ASDA 66%, Chicago 91%). Increases in AHI and proportion diagnosed appeared better explained by changes in scoring methods than key OSA risk factors.

**Conclusion:**

Temporal increases in proportion of females and decreases in socioeconomic status of people undergoing PSG may reflect greater community awareness of sleep disorders. Temporal increases in age and obesity are consistent with secular trends. Changes in scoring methods have major impacts on OSA diagnosis and judgement of disease severity, with important implications for contemporary resourcing of sleep services and interpretation of historical OSA data.

## INTRODUCTION

Obstructive sleep apnea (OSA) is a common, chronic sleep-related breathing disorder characterised by repetitive total or partial collapse of the upper airway during sleep, and has been associated with a wide range of adverse health outcomes^1^. There are well-established, effective treatments for OSA, including weight loss, continuous positive airway pressure (CPAP), mandibular advancement splints, and upper airway surgery^2^.

The prevalence of OSA appears to have increased markedly over the past 40 years. Prior to 1993, OSA was considered a rare medical disorder, primarily affecting males^3,4^. In 1993, the first large community prevalence study in Wisconsin (US) showed OSA (i.e., apnea hypopnea index (AHI)≥5/hr) was common in both males and females, occurring in 24% of males and 9% of females^5^. Updated estimates from the Wisconsin cohort suggest OSA prevalence in the US has increased by up to 55% between 1993 and 2013^6^. Increased prevalence over time is thought to be mainly due to the global obesity epidemic^6^. Another important contributor to higher estimates of OSA prevalence are improvements in the sensitivity of diagnostic methods for OSA over time^7,8^.

Despite a high reported prevalence, widespread health impacts, and availability of safe and effective treatments, a substantial proportion of OSA in the community remains undiagnosed^9,10^. It is, therefore, important to understand factors that influence referral for testing and diagnosis of OSA. Potentially important factors include severity of OSA and awareness about it in the community, access to health care and diagnostic methods used by sleep services. From the perspective of sleep services and health care systems it is particularly useful to understand the characteristics of those undergoing testing and diagnostic sensitivity of tests used in order to tailor services to meet demand. For example, if OSA test diagnosis rates are high then appropriate resources, such as greater provision of CPAP trials, will be required. Understanding these temporal trends is an important consideration in managing existing sleep services, planning new services, and developing future health care messages.

We are unaware of other longitudinal data documenting changes in the characteristics of those tested for and diagnosed with OSA at sleep services over extended time periods. We reviewed our large cohort of consecutive patients who underwent overnight in-laboratory polysomnography (PSG) for suspected sleep disorders at the West Australian Sleep Disorders Research Institute (WASDRI) - a tertiary adult public hospital sleep service located in Perth, Australia^11^. Our aim was to utilise these data to determine long-term temporal trends in: PSG testing, OSA severity, and OSA diagnosis. We also aimed to explore associations with the key OSA risk factors of age, sex, body mass index (BMI), and socioeconomic status.

## MATERIAL AND METHODS

Data has been systematically collected on patients attending WASDRI, as part of an ongoing large-scale cohort study, the Western Australian Sleep Health Study (WASHS)^11^. Referrals to WASDRI were received from medical practitioners throughout Western Australia.

WASDRI patients were assessed by experienced sleep physicians and investigated for sleep disorders, as clinically indicated, with an overnight PSG^11^. This report details data collected since inception on January 01, 1989 to June 30, 2013; the data most recently available for analysis.

### Study population

PSG group:

All consecutive adults (age≥17 years) who underwent diagnostic PSG. If patients required more than one PSG only their initial diagnostic PSG was selected. This group includes those who underwent PSG and were diagnosed with OSA (*OSA sub-group)* and those who did not have OSA *(‘no OSA’ group).* For the purposes of these analyses, in addition to the ‘PSG group’, the ‘*OSA-subgroup’* is of particular interest and is defined below.

OSA sub-group:

“OSA” was defined as AHI [apnea hypopnea index = frequency (apneas + hypopneas)/hour of sleep] ≥5 events/hour on diagnostic PSG.

Diagnosis rate:

The proportion of the PSG group who were diagnosed with OSA.

### Measurements and study variables

During the study period two different PSG scoring rules were used to define AHI:

*Period 1 (January 01, 1989 to July 31, 2002):* the American Sleep Disorders Association (ASDA) definition was used^12^. *Period 2 (August 01, 2002 to June 30, 2013):* the American Academy of Sleep Medicine ‘Chicago’ rules were applied (see supplementary material for detail)^13^.

Respiratory monitoring systems also improved; airflow was monitored by thermistor prior to September 01, 2002 and subsequently by more sensitive nasal pressure cannulae and oronasal thermocouple^7,8^.

Other PSG data analysed: sleep efficiency index (SEI: sleep time/recording time), arousal index (ARI: number cortical arousals/hour sleep) and percentage of study time with peripheral arterial oxygen saturation <90% (T90%).

#### Key OSA risk factors

When patients attended for PSG we recorded age and sex, and measured height (stadiometer) and weight (calibrated scales). BMI was calculated as weight (kg)/[height (m)]^2^. To characterise socioeconomic status we obtained socioeconomic indexes for area (SEIFA) scores calculated by the Australian bureau of statistics (see supplementary material for detail)^14^.

Ethical approval was obtained from the Western Australian Department of Health Human Research Ethics Committee (App ID: 2016/33) and the University of Western Australia (App ID: RA/4/1/8531).

### Statistical analysis

Analyses were performed in R v3.5.1 for Windows^15^.

Summary statistics and comparisons (see Tables 1 and 2)

**Table 1.** Comparisons of key OSA risk factors and PSG characteristics of the cohort according to AHI scoring rule period.

Characteristic	*ASDA*:1989-2002	*Chicago:*2002-2013	p	Effect size¶
**a) PSG group**	n=11,181	n=13,543		
Age (yr)	50 (41-59)	51 (41-62)	<0.001	0.08
BMI	30.1 (26.7-34.6)	31.4 (27.3-36.7)	<0.001	0.10
SEIFA	7 (5-9)	7 (5-9)	<0.001	-0.05
Sex: Male	76.6 (8,561)	61.7 (8,332)		
Sex: Female	23.4 (2,610)	38.3 (5,180)	<0.001	2.04 (OR)
AHI	10.8 (3.2-29.6)	24.3 (11.8-48.1)	Not applicable	
ARI	22.7 (15.3-35.5)	30.6 (20.6-46.7)	<0.001	0.25
SEI	77.7 (66.5-86.1)	75.8 (64.5-84.5)	<0.001	-0.08
T<90% (%)	0.49 (0.04-3.9)	0.58 (0.04-5.35)	<0.001	0.04
**b) OSA sub-group**	n=7,435	n=12,333		
Age (yr)	51 (43-61)	52 (42-62)	0.568	0.01
BMI	31.5 (27.9-36.2)	31.8 (27.8-37.1)	0.037	0.02
SEIFA	7 (5-9)	7 (5-9)	<0.001	-0.04
Sex: Male	80.7 (5,993)	64 (7,876)		
Sex: Female	19.3 (1,436)	36 (4,433)	<0.001	2.35 (OR)
AHI	20.7 (10.6-44.1)	27.4 (14.8-51.5)	Not applicable	
ARI	27.5 (18.5-43)	32.4 (22.3-49)	<0.001	0.14
SEI	76.2 (64.8-85)	75.3 (64.2-84.1)	<0.001	-0.04
T<90% (%)	1.5 (0.27-7.87)	0.78 (0.07-6.38)	<0.001	-0.12

Notes: PSG = Polysomnography; BMI = Body mass index; SEIFA = Socioeconomic index for areas; AHI = Apnea hypopnea index during diagnostic polysomnography; ARI = Arousal index = number of cortical arousals per hour during diagnostic PSG; SEI = Sleep efficiency index = percentage of time asleep during diagnostic PSG; T90% = Percentage of total study time with peripheral arterial oxygen saturation less than 90% during diagnostic PSG; Data presented as medians (quartile 1 – quartile 3) for continuous variables or percentages (numbers) for categorical variables; = Cohen’s d reported for comparisons of age; Cliff ’s delta reported for non-parametric comparisons of other continuous variables; Odds ratios (OR) for categorical variables. Test statistics and effect sizes presented for comparisons between groups.

**Table 2.** Comparisons of key OSA risk factors and polysomnographic variables according to OSA diagnosis.

Characteristic	‘no OSA’ group: AHI<5	OSA sub-group: AHI≥5	p	Effect size
**a) ASDA Period (Jan 1989 – July 2002)**	n=3,533	n=7,435		
Age (yr)	46 (37-55)	51 (43-61)	<0.001	0.43
BMI	27.7 (25.1-31)	31.5 (27.9-36.2)	<0.001	0.40
SEIFA	7 (5-9)	7 (5-9)	<0.001	-0.08
Sex: Male	68 (2,400)	80.7 (5,993)		
Sex: Female	32 (1,129)	19.3 (1,436)	<0.001	0.51 (OR)
AHI	1.6 (0.6-3)	20.7 (10.6-44.1)	Not applicable	
ARI	16.6 (11.62-22)	27.5 (18.5-43)	<0.001	0.62
SEI	80.6 (70.4-88)	76.2 (64.8-85)	<0.001	-0.28
T<90% (%)	0.02 (0-0.14)	1.5 (0.27-7.87)	<0.001	1.43
**b) Chicago Period (Aug 2002 – June 2013)**	n=1,209	n=12,333		
Age (yr)	42 (30-54)	52 (42-62)	<0.001	0.62
BMI	27 (23.8-31.1)	31.8 (27.8-37.1)	<0.001	0.42
SEIFA	7 (5-9)	7 (5-9)	<0.001	-0.14
Sex: Male	37.9 (455)	64 (7,876)		
Sex: Female	62.1 (747)	36 (4,433)	<0.001	0.34 (OR)
AHI	2.9 (1.7-4)	27.4 (14.8-51.5)	Not applicable	
ARI	16.1 (11.5-22.2)	32.4 (22.3-49)	<0.001	1.02
SEI	80.75 (68.6-88.2)	75.3 (64.2-84.1)	<0.001	-0.32
T<90% (%)	0.02 (0-0.15)	0.78 (0.07-6.38)	<0.001	1.04

Notes: BMI = Body mass index; SEIFA = Socioeconomic index for areas; AHI = Apnea hypopnea index during diagnostic polysomnography; ARI = Arousal index = number of cortical arousals per hour during diagnostic PSG; SEI = Sleep efficiency i ndex = percentage of time asleep during diagnostic PSG; T90% = Percentage of total study time with peripheral arterial oxygen saturation less than 90% during diagnostic PSG; Data presented as medians (quartile 1 – quartile 3) for continuous variables or percentages (numbers) for categorical variables; = Cohen’s d reported for comparisons of age; Cliff ’s delta reported for non-parametric comparisons of other continuous variables; Odds ratios (OR) for categorical variables.

Patient characteristics were described by counts and percentages or medians and interquartile ranges (IQRs). Comparisons between groups were made using chi-squared tests (categorical), Student’s t-tests (age) or Mann-Whitney U tests for other continuous variables with skewed distributions. Since two different methods for measuring and scoring AHI were used (i.e., corresponding to ASDA and Chicago scoring periods) most analyses were performed separately for each scoring period. Estimates of effect sizes are presented as Cohen’s *d* for tests of age, Cliff ’s delta^16^ for non-parametric tests of all other continuous variables, or as odds ratios for tests of sex.

Assessing temporal trends in key OSA risk factors (see Table 3) Linear models (LMs) were fitted for analyses of patient age (yr) and SEIFA decile, and generalised linear models (GLMs) were fitted for analyses of BMI and sex. LMs and GLMs were fitted with the single predictor of year, modelled as a continuous variable, and departure of the year coefficient (an estimate of rate of change over time) from zero was tested. The net difference in the mean model-predicted value between the first and last years of each of the ASDA and Chicago scoring periods, ∆, was calculated as an absolute measure of the overall change in each variable over time (i.e., effect size). For modelling of sex as an outcome, odds ratios (95% confidence intervals) were estimated to compare the odds of being female for the last relative to the first year, and as a measure of effect size.

**Table 3.** Year on year trends for OSA risk variables: tests of the effect of Year and net change in model-adjusted means between start and end years of indicated scoring period.

Scoring period	Risk factors	Model	Net change in model-adjusted means		p-value Δ (Year)
			Start year	End year	
**PSG *group***			**1989**	**2013**	
ASDA + Chicago	Age	LM	49.45 (49.04 - 49.86)	51.36 (51.03 - 51.68)	<0.001 **+1.91**
	BMI	GLM (IG)	30.35 (30.15 - 30.56)	33.54 (33.34 - 33.74)	<0.001**+3.19**
	SEIFA	LM	7.14 (7.07-7.22)	6.68 (6.62-6.74)	<0.001 -**0.46**
	Pr (Female)	GLM (Binom.)	0.16 (0.15-0.17)	0.46 (0.45-0.47)	<0.001+**0.30**
			**1989**	**2002**	
ASDA	Age	LM	49.70 (49.10-50.30)	50.13 (49.69-50.58)	0.356+0.43
	BMI	GLM (IG)	30.09 (29.79-30.40)	32.24 (31.99-32.51)	<0.001**+2.15**
	SEIFA	LM	7.39 (7.27-7.50)	6.70 (6.61-6.78)	<0.001**-0.69**
	Pr(Female)	GLM Pr(Female)(Binom.)	0.16 (0.15-0.18)	0.29 (0.28-0.31)	<0.001**+0.13**
			**2002**	**2013**	
Chicago	Age	LM	50.91 (50.39-51.42)	51.14 (50.65-51.63)	0.606+0.23
	BMI	GLM (IG)	31.88 (31.62-32.16)	33.54 (33.25-33.83)	<0.001**+1.66**
	SEIFA	LM	6.81 (6.72-6.90)	6.78 (6.70-6.87)	0.747-0.03
	Pr(Female	GLM Pr(Female(Binom.)	0.31 (0.30-0.33)	0.45 (0.44-0.47)	<0.001**+0.14**
**OSA *sub- group***			**1989**	**2002**	
ASDA	Age	LM	51.23 (50.52-51.93)	52.01 (51.49-52.53)	0.154+0.78
	BMI	GLM (IG)	31.12 (30.76-31.50)	33.96 (33.64-34.30)	<0.001**+2.84**
	SEIFA	LM	7.32 (7.18-7.46)	6.64 (6.54-6.74)	<0.001**-0.68**
	Pr(Female)	GLM (Binom.)	0.13 (0.11-0.14)	0.25 (0.23-0.27)	<0.001**+0.12**
			**2002**	**2013**	
Chicago	Age	LM	51.53 (51.00-52.07)	52.07 (51.56-52.58)	0.250+0.54
	BMI	GLM (IG)	32.21 (31.93-32.50)	34.12 (33.82-34.43)	<0.001**+1.91**
	SEIFA	LM	6.79 (6.70-6.88)	6.74 (6.65-6.83)	0.563-0.05
	Pr(Female)	GLM (Binom.)	0.29 (0.28-0.31)	0.43 (0.41-0.45)	<0.001**+0.14**

BMI: body mass index, SEIFA: Socio-economic index for areas, Pr (Sex=Female): probability of sex being female. ‘IG’ = Inverse Gaussian family GLM with: ‘Binom.’ = Binomial family GLM (with logit link function is logistic regression); Δ = net difference in the model-adjusted means. Estimates in parentheses are the 95% confidence intervals for model-adjusted means.

Multivariable analyses: associations with OSA severity, diagnosis rate (see Table 4)

**Table 4.** Multivariable associations with OSA severity and diagnosis.

Dependent Variable	Patients	Period	Predictor	Statistic	DF	Test statistic value	P	Effect size	R^2^
log AHI	PSG group	ASDA	Year	F	13	12.3	<0.001	0.015	
log AHI	PSG group	ASDA	Sex	F	1	509.3	<0.001	0.026	
log AHI	PSG group	ASDA	Age	F	4	124.7	<0.001	0.039	
log AHI	PSG group	ASDA	BMI	F	4	516.1	<0.001	0.164	
log AHI	PSG group	ASDA	SEIFA	F	4	1.1	0.367	0.000	
log AHI	PSG group	ASDA	Model	F	26	121.8	<0.001		0.243
log AHI	PSG group	Chicago	Year	F	11	18.9	<0.001	0.013	
log AHI	PSG group	Chicago	Sex	F	1	1216	<0.001	0.050	
log AHI	PSG group	Chicago	Age	F	4	160.3	<0.001	0.044	
log AHI	PSG group	Chicago	BMI	F	4	707.4	<0.001	0.169	
log AHI	PSG group	Chicago	SEIFA	F	4	5.3	<0.001	0.001	
log AHI	PSG group	Chicago	Model	F	24	203.3	<0.001		0.277
log AHI	OSA sub-group	ASDA	Year	F	13	2.7	0.001	0.004	
log AHI	OSA-sub-group	ASDA	Sex	F	1	258.2	<0.001	0.009	
log AHI	OSA-sub-group	ASDA	Age	F	4	21.3	<0.001	0.003	
log AHI	OSA sub-group	ASDA	BMI	F	4	294.6	<0.001	0.153	
log AHI	OSA-subgroup	ASDA	SEIFA	F	4	1.4	0.248	0.001	
log AHI	OSA-subgroup	ASDA	Model	F	26	51.9	<0.001		0.166
log AHI	OSA sub-group	Chicago	Year	F	11	9.4	<0.001	0.006	
log AHI	OSA sub-group	Chicago	Sex	F	1	966.3	<0.001	0.032	
log AHI	OSA sub-group	Chicago	Age	F	4	90.1	<0.001	0.019	
log AHI	OSA sub-group	Chicago	BMI	F	4	686.7	<0.001	0.189	
log AHI	OSA sub-group	Chicago	SEIFA	F	4	7.5	<0.001	0.002	
log AHI	OSA sub-group	Chicago	Model	F	24	158.5	<0.001		0.246
OSA diagnosis	PSG group	ASDA	Year	*X^2^*	13	119.4	<0.001	0.96 (OR)	
OSA diagnosis	PSG group	ASDA	Sex	*X^2^*	1	312.9	<0.001	0.36 (OR)	
OSA diagnosis	PSG group	ASDA	Age	*X^2^*	2	433.2	<0.001	1.60 (OR)	
OSA diagnosis	PSG group	ASDA	BMI	*X^2^*	2	901.7	<0.001	2.88 (OR)	
OSA diagnosis	PSG group	ASDA	SEIFA	*X^2^*	2	0.004	0.998	1.00 (OR)	
OSA diagnosis	PSG group	ASDA	Model	*X^2^*	20	1,404.9	<0.001		0.253
OSA diagnosis	PSG group	Chicago	Year	*X^2^*	11	131.2	<0.001	1.21 (OR)	
OSA diagnosis	PSG group	Chicago	Sex	*X^2^*	1	364.7	<0.001	0.26 (OR)	
OSA diagnosis	PSG group	Chicago	Age	*X^2^*	2	355.9	<0.001	1.54 (OR)	
OSA diagnosis	PSG group	Chicago	BMI	*X^2^*	2	408.5	<0.001	2.14 (OR)	
OSA diagnosis	PSG group	Chicago	SEIFA	*X^2^*	2	0.051	0.975	0.98 (OR)	
OSA diagnosis	PSG group	Chicago	Model	*X^2^*	18	1,135.2	<0.001		0.234

Abbreviations - BMI: body mass index, SEIFA: Socio-economic index for areas. Type II ANOVA tests of terms fitted in multivariable models showing the significance of each term, controlling for all others. = adjusted R2 for linear model and the generalised R2 for logistic regression.^18^DF = numerator degrees of freedom for F statistic or degrees of freedom for Wald test (X^2^); denominator degrees of freedom for F tests: 6,618 (OSA patients, ASDA period); 11,550 (OSA patients, Chicago period); 9,774 (All patients, ASDA period); 12,675 (All patients, Chicago period). Effect size = η^2^ (log AHI)^17,37^; odds ratio (OR: AHI≥5) Odds ratios are calculated for the dichotomous variable: male is the referent group for Sex, start year is the referent group for Year (ASDA=1989; Chicago=2002); and for continuous variables (Age, BMI, SEIFA) as mean + 1 SD relative to mean.

Multivariable analyses were undertaken to investigate the associations of OSA risk variables with the diagnosis and severity of OSA in each time period. For analyses of OSA severity, a LM was fitted to the natural logarithm-transformed AHI plus a small constant (0.001; to account for AHI scores of zero). The probability of being diagnosed with OSA (i.e., Pr (AHI≥5)) was modelled using logistic regression. Year of study, as well as patient age, BMI, sex, and SEIFA decile were included as covariates. The significance of covariate associations with OSA severity or Pr (AHI≥5), after controlling for effects of other predictors is shown. Estimates of effect sizes are presented (η^2^)^17^ for continuous variables or as odds ratios for sex (reference level of sex being male). The adjusted *R*^2^ (LMs) or the generalised *R*^2^ (logistic regressions) are also presented^18^.

## RESULTS

**Figure 1.**
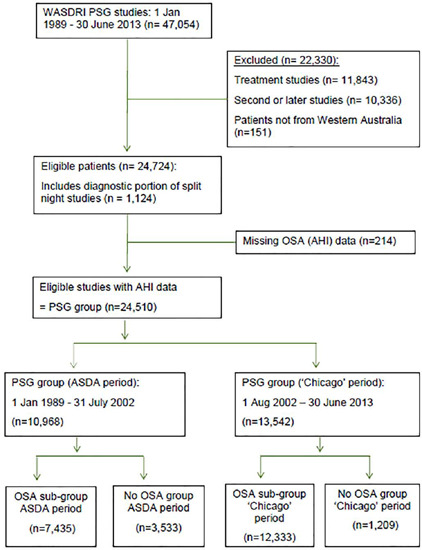
Chart showing selection of sleep studies for analysis from the WASDRI cohort: 1 January 1989 - 30 June 2013.

**Figure 2.**
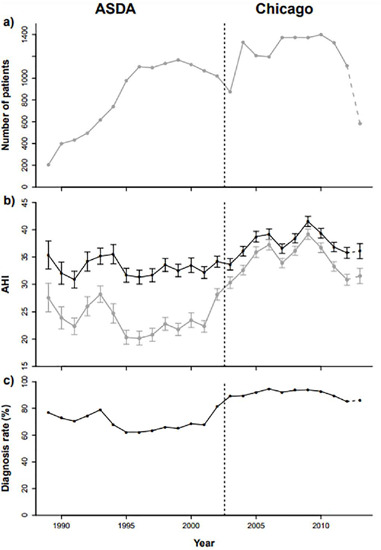
Trends in the WASDRI sleep cohort over time, (a) Numbers of patients undergoing diagnostic PSG (includes those with missing AHI data); (b) Mean AHI (±Standard error) by year; (c) Percentage of patients diagnosed with OSA by year. Figures (b) and (c) exclude those with missing AHI data. Grey plotted values in (b) are for the referred group and black plotted values are for diagnosed (OSA) patients only (AHI=5). Vertical dashed line delineates ASDA from Chicago scoring periods.

Between January 01, 1989 and June 30, 2013, 47,054 patients residing in Western Australia underwent PSG at WASDRI, of whom 24,510 had an initial diagnostic PSG and complete PSG data and form the study population, i.e., the ‘PSG group’ (Figure 1). The number of patients undergoing initial diagnostic PSG increased steadily from 205 in 1989 to 1,104, in 1996, and fluctuated thereafter between a nadir of 875, in 2003, and a zenith of 1,399, in 2010 (Figure 2A).

On average, the study population were predominantly middle-aged, more often male, obese and, by Australian standards, of relatively high socio-economic advantage (median=7^th^ SEIFA decile) (Table 1A). Compared to the ‘no-OSA’ group of patients without OSA, the OSA sub-group were older, more obese, more likely to be male and had lower SEIFA (all p<0.001; Table 2).

### Comparisons between ASDA and Chicago period (summary data):

#### PSG group (Table 1A)

**a) Risk factors**

Compared to the ASDA period, patients undergoing PSG during the Chicago period were more likely to be female (OR=2.04), were more obese (*p*<0.001, effect size of BMI 0.10), were slightly older (*p*<0.001) and had slightly lower SEIFA (*p*<0.001).

**b) PSG sleep variables**

The mean AHI fluctuated from year to year (Figure 2B, grey line). However, median AHI values were 125% higher in the Chicago than the ASDA period. In addition, other PSG metrics during the Chicago period were consistent with increased OSA severity: higher ARI (*p*<0.001) and T90% (*p*<0.001), and lower SEI (*p*<0.001).

#### OSA sub-group (Table 1B)

**a) Risk factors**

Differences in key OSA risk factors between periods in the OSA sub-group were similar to the *PSG group;* however, there was no difference in age between periods.

**b) PSG sleep variables**

Compared to the ASDA period, in the Chicago period, the median AHI was slightly higher (32% higher) and those diagnosed with OSA were categorised as more severe: mild (ASDA vs. Chicago: 38.4 vs. 25.4%), moderate (ASDA vs. Chicago: 25.0 vs. 28.1%) and severe (ASDA vs. Chicago: 36.6 vs. 46.5%). In addition, in the Chicago period, sleep quality as assessed by SEI and ARI was worse but oxygenation, as assessed by T<90%, improved.

### Diagnosis rates

During the study period, 81% of patients who underwent PSG were diagnosed with OSA (i.e., AHI≥5). There was a notable increase in the proportion of patients diagnosed with OSA from the ASDA (68%) to Chicago (91%) time periods (see patient numbers, Tables 1C and 1D). Figure 2C shows the diagnosis rate was relatively stable within each of the ASDA and Chicago periods, but with a consistently higher rate in the latter period.

### Temporal trends in key OSA risk factors:

#### PSG group (Figure 3^,^Table 3)

**Figure 3.**
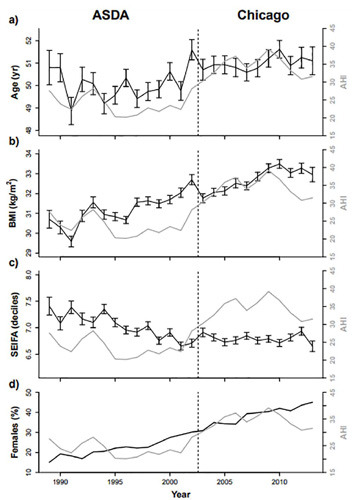
Means of key socio-demographic and anthropometric obstructive sleep apnea risk variables for the PSG group (black lines). Mean AHI shown as grey line for context. Vertical error bars in (a)-(c) show standard errors of means. Percentages of females with year are plotted in (d).

Although mean age varied from year to year with a range of 48.9 to 51.6 years (Figure 3A), there was evidence of a significant linear increase over time (*p*<0.001), with a model-predicted net change from 1989 to 2013 of +1.91 years (Table 3). Similarly, there was evidence of a significant non-linear increase in mean BMI over time, with a net change of +3.19 kg/m^2^ (*p*<0.001; Table 3^,^Figure 3B). Mean SEIFA decile decreased from 7.4 in 1989 to 6.7 in 2001 (ASDA period, *p*<0.001) and remained relatively stable in the Chicago period (*p*=0.747; Figure 3C). The modelling supported an increase in the proportion of female patients over time (Figure 3D and Table 3; *p*<0.001). The average model-predicted increase in the percentage of females from 1989 to 2013 was +30.3%. The odds of being female in 2013, relative to 1989, was 4.57 (95 % CI: 4.10-5.08).

#### OSA sub-group

Trends for BMI, SEIFA and sex in the *OSA sub-group* were similar to the *PSG group* (Table 3; Figure S1 in Supplementary material). However, in contrast to the entire group, there was no temporal trend in age in the OSA sub-group during both ASDA and Chicago periods (Table 3, *p*=0.154 and 0.250, respectively).

### Multivariable associations with OSA severity (Table 4):

#### PSG group

In both ASDA and Chicago periods, there was evidence of a significant log-linear increase in AHI over time (p<0.001) independent of the covariates modelled (Table 4).

In both periods, all the key OSA risk factors were independent predictors of (log-transformed) AHI (all *p*<0.05), with the exception of SEIFA in the ASDA time period (*p*=0.37; Table 4). However, estimated effect sizes (η^2^) were relatively small for all factors except BMI (η^2^=67% and 61% of unadjusted *R*^2^ in ASDA and Chicago period, respectively). The estimated effect sizes for time were small in both the ASDA and Chicago periods (η^2^=6.1% and 4.7% of unadjusted *R*2 in ASDA and Chicago period, respectively).

In both periods, the model-adjusted back-transformed median AHI was lower in females than males, after adjusting for other risk factors (female vs. male: ASDA 6.9 vs. 11.4/h; Chicago 16.5 vs. 30.6/h) (data not shown).

#### OSA sub-group

Inferences derived from multivariable modelling of OSA severity in the *OSA sub-group* were similar to those of the *PSG group* (Table 4).

Multivariable associations with OSA diagnosis (Table 4): All OSA risk factors were significant predictors of the risk of being diagnosed with OSA (i.e., Pr (AHI≥5)) independent of the covariates investigated (*p*<0.05), except for SEIFA (Table 4). For both ASDA and Chicago periods, the strongest predictor of probability of OSA diagnosis was increasing obesity followed by male sex and increasing age. Time (‘year’) had a weak influence (Odds ratio ASDA = 0.96, odds ratio Chicago = 1.21) and SEIFA had no significant effect (Table 4).

## DISCUSSION

We investigated temporal patterns in the testing for and diagnosis of OSA and in key OSA-associated risk factors over a 23.5 year period in a major adult tertiary sleep service. The study period encompasses the early days of systematic PSG investigation for sleep disorders until the use of contemporary techniques. We are unaware of previous publications with similar long-term longitudinal data from large sleep clinic cohorts. Given the potential relevance of such data to health care planning and to interpretation of long-term epidemiological data, we now describe these trends in our service. It appears likely that the data are reflective of international trends as: (a) international PSG diagnostic methods and scoring rules were used with changes in their nature made contemporaneously with other centers around the world; (b) changing obesity rates are consistent with those described elsewhere; and (c) the growing proportion of diagnosed OSA over the course of the study reflects the growing proportions of OSA observed in longitudinal cohort studies over a similar period^5,6^.

### Key OSA risk factors

One of the most notable findings was the increasing proportion of females undergoing PSG. Firstly, summary data show increase odds of females undergoing PSG in the Chicago compared to the ASDA period. Secondly, the graphical and modelling data confirm a steady and progressive, almost threefold, increase in the percentage of females undergoing PSG (from 15.1% in 1989 to 45.0% in 2013). The female:male ratio in Western Australia (all ages) decreased slightly, between 1996 (1:1.011) and 2016 (1:1.1014)^19^, i.e., in the opposite direction to these clinic changes. This finding suggests there was increasing community awareness over time about the importance of OSA as a cause of symptoms in females. In the early days of sleep medicine OSA was considered to be a rare condition in females, with an estimated female:male ratio of approximately 1:10^3^. However, these estimates were based on clinic samples and more recent community-based estimates show that, the sex ratio is closer to 1:2^20^.

There was a small increase in BMI and a marginal increase in age of those undergoing PSG. Australian Nutrition survey data showed the Australian age standardised percentage of adults who were overweight (25≥BMI≤30kg/m^2^) or obese (BMI>30kg/m^2^) increased from 64.9 to 69.9% among males and from 49.4 to 55.2% among females between 1995 and 2011^21^. In Western Australia, the median age increased from 33 to 36.2 years and the proportion of adults aged ≥65 years increased from 10.5% to 12.2% between 1996 and 2011^19^. Hence, the changes we found in BMI and age in those undergoing PSG are likely to reflect secular increases in obesity and longevity in the general community. Socioeconomic status of those undergoing PSG declined slightly between 1989 and 2001, most likely reflecting increased community awareness of the importance of sleep disorders, and then did not change significantly.

**Figure S1.**
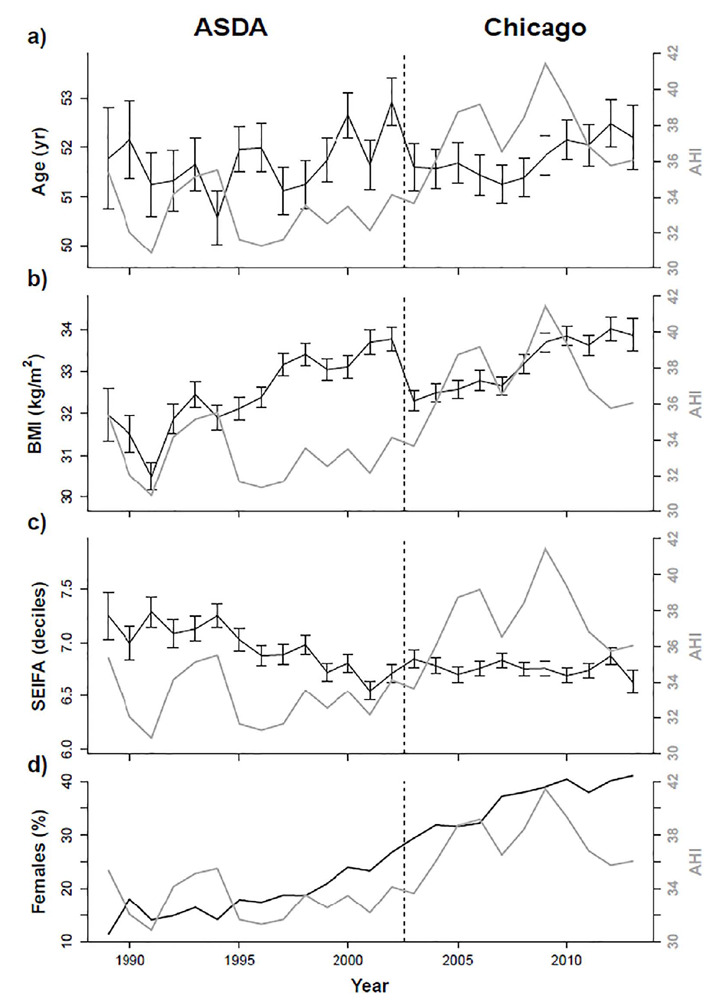
Means of key sociodemographic variables for patients diagnosed with OSA (AHI ≥ 5) (black lines) with mean AHI shown as grey line for context. Vertical error bars in (a)-(c) show standard errors of means. Percentages of females with year are plotted in (d).

Those diagnosed with OSA showed broadly similar temporal trends in OSA risk factors to the patterns seen in those undergoing PSG (Supplementary Figure S1 and Table 2).

### OSA severity

In both the PSG group and OSA subgroup the effect of year on the AHI trend within each scoring period was small. These small increases in AHI within periods could be partly explained by changes in key OSA risk factors (i.e., age, sex, and BMI). Our multivariable predictive model indicated that BMI was the variable most closely associated with AHI in each temporal period. So, for example, in the PSG group the mean patient BMI increased by an estimated 3.19kg/m^2^ over the study period, and a concomitant increase in AHI may be expected. Although not directly comparable, bariatric surgical studies suggest that a change in BMI of this magnitude would be associated with a modest AHI increase of 7-8/hr^22^. Increasing age has consistently been associated with increased AHI and more severe OSA^23^. However, females presenting to sleep clinics usually have a lower AHI than males^24,25^, including when matched for BMI^26^. Consistent with those studies, we found that adjusted mean AHI was lower for females compared to males. Hence, the temporal trends in sex ratio may have offset the impact of an increase in BMI and age. We did not find an independent predictive role for socioeconomic status in our OSA severity model, which is broadly consistent with previous studies^27,28^. Our data suggest the global trends of increasing obesity and ageing, particularly in developed countries, are likely to lead to more severe OSA cases referred to sleep clinics, in the absence of offsetting factors, such as increases in the percentage of referred females.

In contrast to the small changes within scoring periods, the changes in mean AHI between periods, as shown by summary data for each period, were relatively large. In the PSG group there was a marked (125%) increase and in the OSA subgroup a more modest (32%) increase in mean AHI from the ASDA to the Chicago periods. The magnitude of the increase in AHI between periods is out of proportion to the relatively small changes observed in other PSG measures of OSA severity and in key OSA risk factors (sex, age, and BMI), as discussed above. Other recognised OSA risk factors including central obesity^29^, physical activity^30^, and craniofacial restriction (e.g., secondary to ethnic differences^31^) w ere not measured in the current study and could contribute to the changes in AHI/OSA severity between the study periods. Nevertheless, the changes in scoring rule and measurement sensitivity are known to have a sizeable effect on estimated AHI7,^8,32^ and are the most likely explanations for the above increases in mean AHI (and the shift to more severe categories of OSA) between the ASDA and Chicago periods. Of note, overnight oxygen levels in the OSA sub-group improved in the latter Chicago period, compared to the earlier period. This reflects the lower oxygen desaturation threshold (≥3% or no desaturation criterion when cortical arousals are associated with events) for scoring hypopneas during the Chicago compared to the ASDA (≥4% oxygen desaturation) period.

### OSA diagnosis rates

A striking finding in the summary data was the much higher diagnosis rate in the Chicago (91%) compared to the ASDA (68%) period, most likely related to changes in scoring rules and sensitivity of measurement methods. Figure 2C shows relatively flat diagnostic rates within each period and an apparent step change upwards, around 2002. Consistent with these data, the trend model shows a lack of change in diagnosis rates within each scoring period. In particular, after adjusting for OSA risk factors, the 95% confidence interval for the odds ratio for being diagnosed, to not diagnosed, for the latest year relative to the earliest (reference year) in each period overlapped 1 (ASDA period OR: 0.96, 95% CI: 0.57-1.62; Chicago period OR: 1.21, 95% CI: 0.79-1.86) (data not shown).

Despite the large increases over time in the proportion of females undergoing PSG our multivariable modelling showed that the odds of a female being diagnosed with OSA (after controlling for other co-variates) were low relative to the odds for males. This difference may reflect previously described differences in symptom profile between sexes. Males tend to have more ‘classical’ OSA symptoms of loud snoring and sleepiness, whereas females have more fatigue, depression, and insomnia symptoms^33,34,35^. The symptoms experienced by females may be more numerous and less specific which can widen the differential diagnosis^36^, and so referring physicians may be less accurate in their clinical assessment when assessing females. As a corollary, a greater number of females, compared to males, may have OSA in the differential diagnosis, leading to higher proportion referred for assessment which might explain the relatively high female:male ratio (1:1.2) among those referred to our clinic by 2013. These data suggest that screening methods tailored to characteristic features of OSA in females may be useful in reducing the need for PSG evaluation and its associated costs.

### Limitations

We do not have data on referral patterns for OSA diagnosis in the wider community and so cannot provide direct information on these trends. However, we believe the data on those undergoing PSG in our cohort are likely to be reflective of such referral patterns in the broader West Australian community, for the following reasons. Firstly, WASDRI was the only public sleep clinic serving the state of Western Australia during much of this period, except from July 2007 onwards when a small second public sleep service commenced, providing an average of 200 PSGs per year. However, the increased PSG capacity provided by private sleep services that evolved during the study period is not known. Secondly, diagnostic PSGs were performed on nearly all patients referred to WASDRI clinic during the study period. Medical record review of a random sample of 200 WASDRI referrals during the study period indicated 90.5% underwent PSG and there were no significant differences in age, sex, or BMI between patients undergoing PSG and those not undergoing PSG (data not shown, all p>0.05).

We do not have data on the reasons given by physicians for referral. However, OSA is commonly considered in the assessment of possible causes for sleep-related symptoms, for example as an aggravating comorbidity in conditions such as insomnia, parasomnias, and narcolepsy. Diagnosis rates in this study need to be considered in the above context. We did not attempt to exclude those with predominant central sleep apnea (CSA), however in our cohort a small proportion had predominant CSA (2.0%) as defined by CSA index >5/hour and central apnea index >50% of obstructive apnea index.

## CONCLUSION

This study highlights the substantial and steady increase in the proportion of females undergoing PSG and receiving a diagnosis of OSA in a large sleep disorder service over almost a quarter of a century, until recent years. This pattern likely reflects increasing community awareness of OSA in females. However, females presenting to sleep services have lower odds of being diagnosed, compared to males, and tailored screening methods to better identify OSA in females may be useful. There has also been a small increase in the obesity levels and age of patients referred, which may reflect increasing obesity and ageing in the community. BMI was the strongest predictor of AHI in a multivariable model hence, if these trends continue, the severity of OSA referred to sleep services will increase.

While it is difficult to be certain about the effect of changes in scoring rules (and measurement techniques) in an observational study these real-world data suggest such changes can have significant impacts on diagnosis rates, characteristics of those diagnosed and their reported disease severity (AHI). The magnitude of these changes highlights the need for careful evaluation of OSA literature from different scoring eras. There are important resource implications for health service delivery when changes in diagnostic methods lead to higher diagnosis rates. There is a need to carefully evaluate whether associated higher diagnosis rates lead to improved health outcomes.

## Figures and Tables

**Figure S2.**
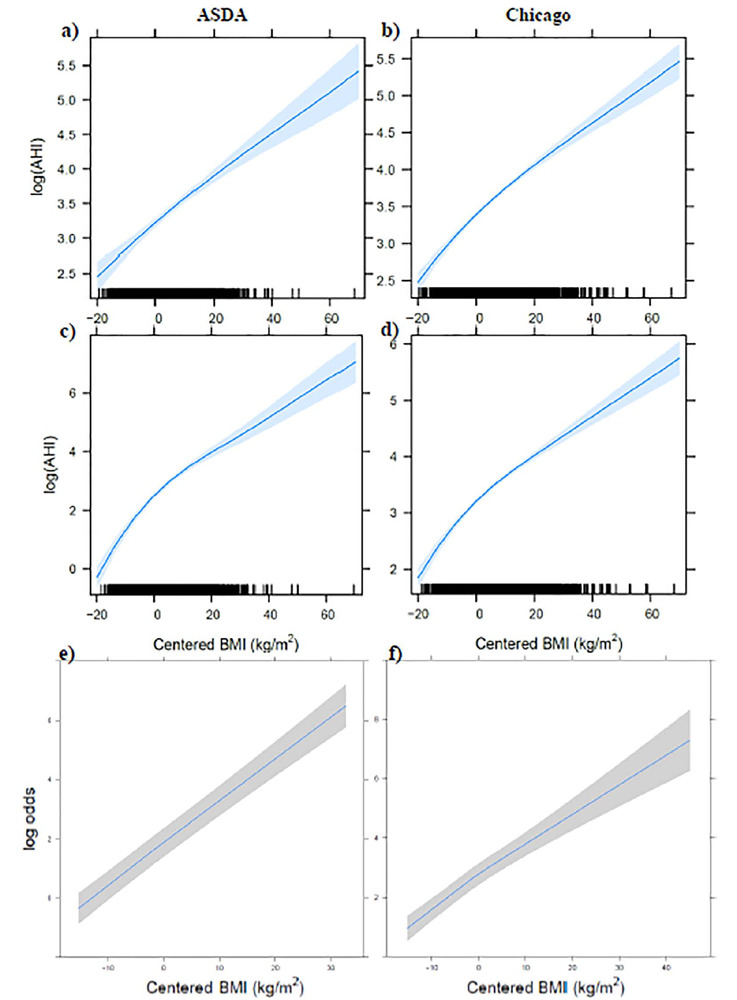
Marginal effects plots showing the model-adjusted association of OSA severity (log AHI) for OSA patients (OSA-subgroup) (a, b), PSG group patients (c, d) and the log odds of OSA diagnosis (AHI ≥ 5) (e, f), with body mass index (BMI).

## SUPPLEMENTARY DATA FILE:

The changing profile of obstructive sleep apnea: long term trends in characteristics of patients presenting for diagnostic polysomnography

**1. Figure S1:** Additional plots for year-on-year trends in patient characteristics: OSA-sub group patients.

**1. Figure S2:** Additional plots for relationships with OSA severity (multivariable analyses).

## MEASUREMENTS AND STUDY VARIABLES

**PSG scoring rules:**

During the study period two different PSG scoring rules were used to define AHI:

*Period 1 (1 January 1989 to 31 July 2002):* the American Sleep Disorders Association (ASDA) definition was used.(“Indications and standards for cardiopulmonary sleep studies. American Thoracic Society. Medical Section of the American Lung Association,” 1989) In brief, apneas were scored when there was a reduction in oronasal flow (using thermistor) >90% from baseline. Hypopneas were defined by a reduction of airflow (using thermistor) >50% of baseline flow in association with an oxygen desaturation ≥4% (oximeter).

*Period 2 (1 August 2002 to 30 June 2013):* the American Academy of Sleep Medicine ‘Chicago’ rules were applied.(“Sleep-related breathing disorders in adults: recommendations for syndrome definition and measurement techniques in clinical research. The Report of an American Academy of Sleep Medicine Task Force,” 1999) In brief, there was no change in the apnea rule, however the hypopnea definition changed to a reduction of airflow >50% of baseline flow or a clearly perceptible but lesser reduction in flow associated with either a cortical arousal or an oxygen desaturation of ≥3%. Apneas and hypopneas required a duration ≥10 seconds in both scoring rules.

**Key OSA risk factors:**

To characterise socio-economic status we obtained Socio-Economic Indexes for Area (SEIFA) scores calculated by the Australian Bureau of Statistics (see supplemental material for detail).(Australian Bureau of Statistics, 2016) The SEIFA score used was an Index of Relative Socio-Economic Advantage and Disadvantage, and we used the deciles of SEIFA calculated for individual suburbs, from the distribution of all Australian suburbs.(Australian Bureau of Statistics, 2016) SEIFA deciles for de-identified individuals were obtained by matching the postal code for their residential suburb to postal codes corresponding to SEIFA deciles.

*Statistical analysis – supplementary information:*

*Summary statistics and comparisons (see Table 1 and 2)*

Patient characteristics were described by counts and percentages or medians and interquartile ranges (IQRs). Comparisons between groups were made using Chi-squared tests (categorical), Student’s t-tests (age) or Mann-Whitney U tests for other continuous variables with skewed distributions. Since two different methods for measuring and scoring AHI were used (i.e., corresponding to ASDA and Chicago scoring periods) most analyses were performed separately for each scoring period.

Comparisons of sex (categorical) between groups was performed using Chi-squared tests of independence, and Student’s t-tests were used to compare group means of age (continuous), which demonstrated negligible skew (Skewness=0.016). Non-parametric Mann-Whitney U tests of location were used to compare groups for other continuous variables with skewed distributions (ARI, BMI, SEI, T<90% (%)) and SEIFA deciles, which had been calculated from ordinal scores.

*Assessing temporal trends in key OSA risk variables (see Table 3)*

Generalised Linear Models (GLMs) were fitted for analyses of BMI (kg/m^2^) using Inverse Gaussian GLM with reciprocal link function and for sex using Binomial GLM with logit link function.

The Box-Cox method was used to select a suitable transformation of the response variable for linear modelling (i.e., taking the natural logarithm of AHI) or a suitable distribution for modelling the response variable using a GLM.(1) For these analyses, LMs and GLMs were fitted with the single predictor of Year, modelled as a continuous variable. Departure of the year coefficient (an estimate of rate of change over time) from zero was tested using either a Student’s *t*-test, or, for the Sex GLM, using a difference in model deviances test, which compares nested models with and without the year term.(2)

*Multivariable analyses: associations with OSA severity, diagnosis rate*

In the analyses continuous variables (age, BMI, SEIFA) were mean-centred prior to modelling and then modelled using natural splines, with linear boundary constraints past the 10^th^ and 90^th^ percentiles, to account for non-linear relationships.

1. Szentkiralyi A, Fendrich K, Hoffmann W, Happe S, Berger K. Socio-economic risk factors for incident restless legs syndrome in the general population. J Sleep Res. 2012;21(5):561-8.

2. Faraway JJ. Extending the Linear Model with R. Boca Raton: Chapman & Hall/CRC; 2006.
